# Providing medical care in unfamiliar settings; experience of an Egyptian campaign in Uganda

**DOI:** 10.11604/pamj.2014.17.111.3728

**Published:** 2014-02-14

**Authors:** Ahmed Hasanin, Nadine Sherif, Mohamed Elbarbary, Doaa Mansor

**Affiliations:** 1 Faculty of medicine, Cairo University, Egypt

**Keywords:** Surgical camp, Africa, Uganda

## Abstract

Medical service in many African countries is affected by the limited infrastructure and the lack of economic and human potentials. Uganda is one the countries that suffers from lack of physicians as well as shortage of many medical facilities with many endemic health problems such as Goiter. A surgical camp was done by an Egyptian team of 8 physicians; three general surgeons, one pediatric surgeon, two gynecologists and one anesthetist. Over two hundred cases were screened in the outpatient clinic. Eighty nine operations were done in four days. General surgery procedures were 45 operations (50%), Pediatric procedures were 23 operations (26%) and Gynecological operations were 21 operations (24%) In conclusion Humanitarian relief for poor population in the developing world countries needs vigilant international collaboration. Special attention should be given to goiter in African countries. Training doctors from sub-Saharan African nations should be on the top of the agenda of the international medical community in order to reach a definitive solution for their health problems.

## Introduction

Medical service in many African countries is affected by the limited infrastructure and the lack of economic and human potentials. Few numbers of physicians are present with marked shortage of some specialties that give limited chances for proper treatment of patients even in minor health problems.

Uganda is one the countries that suffers from lack of physicians as well as shortage of many medical facilities. Approximately there is one doctor per 20000 sick people, thus many people go untreated. Uganda is a landlocked country in East Africa it is also known as the “Pearl of Africa”. It is bordered Kenya, South Sudan, Rwanda, Tanzania, and Democratic Republic of the Congo.

Surgical camps have been one of these initiatives performed by many countries and committee to many African countries aiming to; provide a good service to the largest possible number of patients, train the present physicians at the country, explore and clarify the extent of the current health problems to the international community.

We previously described our experience in working under unfamiliar circumstances during the Egyptian revolution [1]. Here we discuss our experience in a four day camp to Kampala (Ugandan Capital).

**Figure 1:**
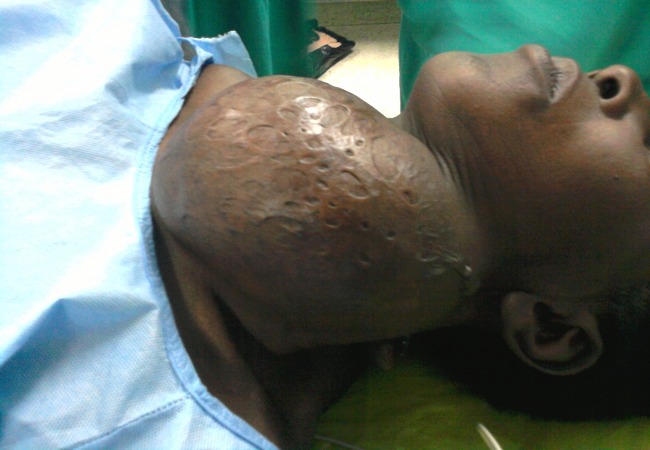
Huge goiter

**Figure 2:**
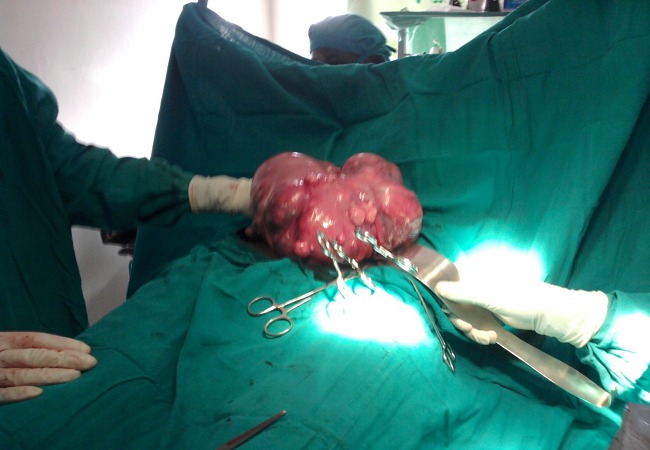
A case of multiple uterine myomas

**Endemic health problems in Uganda:** Goiter is a major health problem in many African countries including Uganda due to environmental and nutritional factors. Prevalence of goiter (Figure 1) reached 30% in some areas due to dietary iodine and selenium deficiency [2, 3]. Also uterine fibroid (Figure 2) is another common health problem among the Africans [4, 5]. Thus most cases managed during our short camp were focused on these two disorders (Goiter and uterine fibroid) in addition to pediatric cases

**Figure 3:**
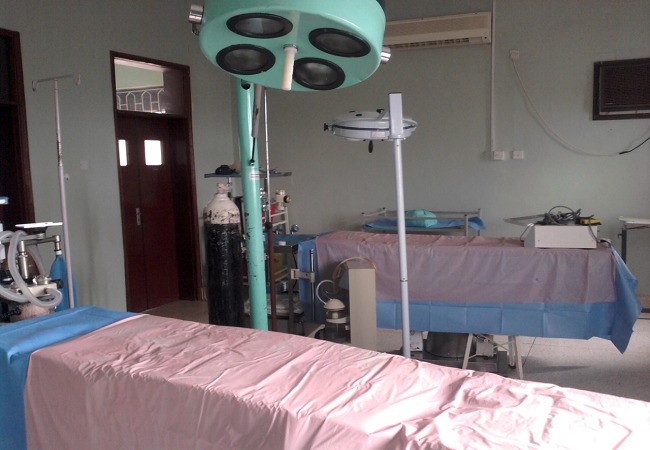
Operating room with two operating tables

**Location (Kibuli hospital):** The surgical camp was at Kibuli hospital; a charity hospital located on Kibuli hill in the southeastern part of Kampala. It was founded as an outpatient facility at 1975, added 59 beds in 1995, and finally reached 200 beds. It has a blood bank, 3 operating rooms with 5 operating tables, and a laboratory. However it had a few number of physicians and no ICU beds. (Figure 3)

**The surgical team:** The team consisted of 8 physicians; three general surgeons, 1 pediatric surgeon, 2 gynecologists and one anesthetist. Over two hundred cases were screened in the outpatient clinic; Eighty nine operations of different specialties were done in 4 successive days. All physicians were volunteers, all medical and surgical services provided were free.

## What we accomplished

**Procedures:** General surgery procedures were the most frequent operations with total number 45 operations (50%), general surgery procedures included; Hemithyroidectomies (27 cases), Total Thyroidectomies (4 cases), Hernias (13 cases), and open cholecystectomy (one case). Pediatric procedures reached 23 operations (26%) and included hernias (19 cases), Hypospadius repair (3 cases) and undescended testis (one case). Gynecological operations reached 21 operations (24%) most of them were Hysterectomies (10 cases), Myomectomies (6 cases), Classical repair (two cases), Ovarian Cystectomy (one case), Vulval mass excision (one case), and drainage of pyosalpinx (one case) (Table 1).

**Table 1:** Surgical interventions (number, %)

General surgery	Number of operations
Hemithyroidectomy	27
Total thyroidectomy	4
Oblique inguinal hernia	12
Epigastric hernia	1
Open Cholecystectomy	1
**Total**	**45 (50%)**
Pediatric surgery	
Oblique inguinal hernia	15
Paraumbilical hernia	4
Hypospadias	3
Undescended testis	1
**Total**	**23 (26%)**
Gynecological operations	
Abdominal Hysterectomy	8
Vaginal hysterectomy	2
Myomectomy	6
Classical repair	2
Ovarian cystectomy	1
Vulval mass excision	1
Drainage of pyosalpinx	1
**Total**	**21 (24%)**

**Adverse events:** Few adverse events were experienced during the camp with no effect on the final outcome. Repeated break in the electric power was an evident problem. Oxygen cylinders had nonfunctioning pressure gauges, this resulted in repeated emptying of the cylinders during the operations without alarm, and this needed close monitoring to the gas flowmeters to detect any decrease in oxygen flow. A case of massive post-operative bleeding after myomectomy operation needed urgent exploration that revealed massive oozing from the surgical bed. The patient done total abdominal hysterectomy and was packed for 24 hours. The patient received 8 units of whole blood and 10 units of fresh frozen plasma. Packs were removed after 24 hours with no additional sequelae.

## Discussion

**Surgical challenges:** Many challenges have faced the surgeons because of the lack of equipment, difficulties in communication, lack of assistants and shortage of medical supplies. Very poor light sources were present, only one diathermy and a vessel sealing device (LigaSure™) delivered with the team from Egypt. Another challenge was the need for screening and operating a large number of cases in a very short time by a few number of physicians, this needed extra ordinary effort from all the team members working long hours with few breaks.

For pelvic surgeries, the high rate of PID (Pelvic Inflammatory diseases) [5] made the pelvic surgeries challenging due to the extensive adhesions present, which reached in some cases to frozen pelvis.

Another challenge for the surgeons was the prevalence of HIV AIDS which reached more than 10% of the adult population in Uganda. This made screening for HIV AIDS a routine investigation for all patients. Five patients (5.5%) of those needed surgical intervention were HIV positive.

**Anesthetic challenges:** There were five operating table in three operating rooms with variable levels of equipment. Four anesthesia machines were present with only two ventilators (Figure 3).

The term “Bagging” is a known term there which means manually ventilating the patient under general anesthesia by an anesthesia nurse when the anesthesia machine has no ventilator. Bagging was frequently used during our convoy. Gas cylinders were the only source of medical gases as there was no pipeline, only one table had a monitor with non-invasive blood pressure monitoring, pulse oximetry and ECG but there were no ECG electrodes available. Monitoring for other operating tables was done using a portable pulse oximeters and manual blood pressure monitors. These limited monitoring facilities added another burden to the management of anesthesia. Another obstacle was the availability of one anesthetist which was compensated by the aid of anesthesia nurses who are well trained for monitoring. The presence of these nurses helped to operate 5 operating rooms at the same time. Most cases received spinal anesthesia except for thyroidectomy operations this helped to overcome the relative deficiency of anesthetists and the shortage in ventilators.

**Drugs and intravenous fluids**: Most basic anesthetic agents were present however muscle relaxants were relatively deficient. We used propofol, thiopental in induction of anesthesia. Both Halothane, and Isoflurane were used in maintenance of anesthesia. Most cases received atracurium or cisatracurium except cases of suspected difficult intubation (e.g. huge goiters) who received suxamethonium in induction of anesthesia. The only vasopressor present was epinephrine.

**Airway management:** There were no airway devices except endotracheal tubes, oral airways, and face-masks. Laryngeal mask airways were delivered with the team from Egypt. Huge goiters (Figure 1) were challenging in their airway management however there were only two choices either to manage the cases with the available resources or to leave the patients untreated.

**Postoperative pain:** Due to the lack of post-operative monitoring facilities and the large number of operations done in a limited period, there was no proper post-operative pain management except non-steroidal anti-inflammatory agents. An interesting finding was that most patients in this country showed high pain tolerance with minimal complaints regarding post-operative pain.

**Communication obstacles:** As “Swahili” is the main popular language in Uganda followed by English language, communication between the members of the Egyptian team with the Ugandan medical personnel faced many barriers especially because even those who speak English among the Ugandans had their special accent. Of course the communication with the patients during screening was much more difficult as most of them don′t speak English at all. Another communication problem was that most of the personnel were not trained well for surgical assistance and they even don't know well the name of instruments. Another problem was the brand names of drugs which are different from what the physicians were accustomed to in Egypt.

**Similar experience:** Comparing our experience in working in unfamiliar environment during the Egyptian revolution on January 2011 [6] with our experience in this surgical camp; we assume that the main difficulties during the Egyptian revolution was that large number of injured in a very short time and the shortage of medical personnel due to security issues, but we had the advantage of working in a well-equipped tertiary hospital with all facilities. In our camp there were no trauma patients and the rate of cases was more organized but the main disadvantage was working with limited equipment and in unfamiliar settings with many language barriers.

Another article describing the anesthetic practice in Haiti after the 2010 earthquake assumed many similar challenges as we faced in our camp such as communication and language barriers, shortage of medications, and large number of patients but our camp differed in the type of procedures [7].

Poor available resources made the team work with a known logic of “do the best for the most” and not “everything for everyone”. We aimed to bring the best possible care not the best care as described in the academic literature. This is well known in mass causalities and war surgery [8].

**Key messages and lessons:** In future camps we recommend focusing on simple procedures that can be done in small hospitals with limited facilities. We also recommend paying more attention to endemic problems such as Goiter and Vesico-vaginal fistulae. Good preparation needs communication with the health committee in the country to prepare the patients and the equipment and Communication with colleagues who previously visited these areas to obtain information about the facilities. We also recommend accompanying nursing personnel in future camps to facilitate proper assistance and communication. Taking some instruments and drugs with the camp is also essential. Training doctors from sub-Saharan African nations should be on the agenda of the international medical community in order to reach a definitive solution for their health problems.

## Conclusion

Humanitarian relief for poor population in the developing world countries needs vigilant international collaboration. More attention to African countries, especially countries of river Nile, should be paid by Egyptian society.
